# Strategic Motives Drive Proposers to Offer Fairly in Ultimatum Games: An fMRI Study

**DOI:** 10.1038/s41598-017-00608-8

**Published:** 2017-04-03

**Authors:** Yin-Hua Chen, Ying-Chun Chen, Wen-Jui Kuo, Kamhon Kan, C. C. Yang, Nai-Shing Yen

**Affiliations:** 1 Research Center for Mind, Brain, and Learning, National Chengchi University, Taiwan, No. 64, ZhiNan Road, Sec. 2, Taipei, 116 Taiwan; 2 Department of Psychology, National Chengchi University, Taiwan, No. 64, ZhiNan Road, Sec. 2, Taipei, 116 Taiwan; 3 Institute of Neuroscience, National Yang-Ming University, Taiwan, No. 155, Sec. 2, Taipei, 112 Taiwan; 4 Institute of Economics, Academia Sinica, Taiwan, No. 128, Sec. 2, Academia Road, Taipei, 115 Taiwan; 5 Department of Public Finance, National Chengchi University, Taiwan, No. 64, ZhiNan Road, Sec. 2, Taipei, 116 Taiwan; 6 Department of Public Finance, Feng Chia University, Taiwan, No. 100, Wenhwa Road, Seatwen, Taichung 407 Taiwan

## Abstract

The hypothesis of strategic motives postulates that offering fairly in the Ultimatum Game (UG) is to avoid rejection and receive money. In this fMRI study, we used a modified UG to elucidate how proposers reached decisions of offering fairly and to what extent they considered offering selfishly with different stakes. We had proposers choose between a fair and a selfish offer with different degrees of selfishness and stake sizes. Proposers were less likely and spent more time choosing the fair offer over a slightly-selfish offer than a very selfish offer independent of stakes. Such choices evoked greater activation in the dorsal anterior cingulate cortices that typically involve in allocation of cognitive control for cost/benefit decision making. Choosing a fair offer in higher stakes evoked greater activation in the anterior cingulate gyrus (ACCg) and the areas that previously have been implicated in reward and theory of mind. Furthermore, choosing a slightly selfish offer over a fair offer evoked greater activation in the anterior cingulate sulcus, ACCg, ventral tegmental area (or substantia nigra) and anterior insular cortex signalling the higher gain and implying higher rejection risk. In conclusion, our findings favoured the hypothesis that proposers offer fairly based on the strategic motives.

## Introduction

Consider a bargaining environment in which there is a proposer, a recipient and a certain amount of money (i.e. stakes) to be divided between two players. The proposer proposes how to share the sum and the recipient can either accept or reject the proposal. If the recipient accepts the proposal, the money is divided as proposed, whereas both players receive nothing in the case of rejection. This game is known as the ultimatum game (UG)^1^. Although simple, it is important because it has many real-world analogies (such as wage bargaining between a union and the CEO) and helps crystallize more complex bargaining situations.

According to the standard economic theory of self-interest, the recipient accepts any offer greater than zero, and the proposer offers the lowest amount possible^2^. However, experimental studies have provided robust evidence that recipients and proposers take actions that are inconsistent with the theoretical prediction. The majority of proposers share equally and offer approximately 40% of the stakes on average, whereas recipients routinely reject offers ≤20% of the stakes^3–6^. Two hypotheses have been proposed to explain why proposers distribute money in a relatively fair manner^6, 7^.

The first hypothesis suggests that proposers care about the welfare of others and behave generously out of altruistic motives^6, 8–10^. Previous studies examined this hypothesis by comparing the proposing behaviours between the UG and the dictator game (DG)^6, 11–14^, in which the recipient can merely accept the proposal^9, 15, 16^. This hypothesis predicts that proposers would also offer fairly in the DG. However, proposers were found to offer less in the DG (i.e. approximately 23% of the stakes) than that in the UG.

Only two recent studies have examined the neural correlates of the proposing behaviours between the UG and the DG using functional magnetic resonance imaging (fMRI)^13, 14^. Neither study used the original UG task, in which proposers can freely indicate their offer. Instead, they asked participants to choose one preferred offer among several (i.e. 6:6, 7:5, 8:4, 9:3, 10:2 or 11:1 in splitting 12 cents; see ref. 13) or to choose between a fair offer and a selfish offer with different degrees of selfishness (i.e. ¥5: ¥5 vs. ¥7: ¥3 or ¥8: ¥2; see ref. 14). Weiland *et al*.^13^ reported that fair offers in the UG induce greater activation in the striatum than that of the DG, which is involved in reward expectancy and magnitude, suggesting that UG proposers are mainly driven by egotistic motives that emphasize reward. Zheng and Zhu^14^ reported that the same contrast induces greater activation in the right superior temporal gyrus (STG) and left cingulate gyrus. These two areas have been found to engage in making inferences about other people’s mental state and emotional experiences, respectively^15^, suggesting that fair UG offers may be the results of inferring the recipient’s responses.

Strategic motives are the second main hypothesis, postulating that a fair offer is made to avoid the possibility of rejection and receive a monetary reward^8, 17, 18^. This hypothesis has been used to explain why raising stakes has no marked effect on the proposing behaviour^3, 19–21^. That is, offering fairly, regardless of the stakes, is the safest solution in which the possibility of rejection is almost zero and proposers will be guaranteed half the stakes. In cases where proposers were explicitly informed that recipients would receive any offer greater than zero, they offered much less (i.e. 25% of the stakes) and even less when the stakes were raised^22^. In other words, proposers made an offer mainly driven after deliberating on the recipient’s answer.

One important limitation in most previous studies was that only the final proposed offer was observed: participants were asked to divide the money by freely indicating their proposal (e.g. refs 1, 5, 23) or choosing one preferred offer among several offers with different share sizes, such as offering 20%, 30% or 50% of the stakes to the recipient (e.g. refs 11, 13, 24). However, how proposers reached the final decision of ‘offering fairly’ and to what extent they considered a selfish offer remains poorly understood. To tackle this issue, we followed Zheng and Zhu^14^ by using a modified UG in which proposers had to make a binary choice between a fair offer and a selfish offer. Specifically, we manipulated the share size of the selfish offer systematically to be 10%, 20%, 30% or 40% of the stakes rather than either 20% or 30% of the stakes as tested by Zheng and Zhu^14^. This manipulation gave us the opportunity to approximate the threshold of a selfish offer that proposers might have considered even though they ended up making a fair offer. We also manipulated stake size to investigate whether raising the stake size would have no marked effect in our modified UG as reported for the classical UG (e.g. ref. 3). Importantly, we conducted the experiment using fMRI and measured the time that proposers needed to make a choice. Therefore, we were able to examine the UG proposing behaviour at various levels, including the decisions (fair/selfish offers), reaction times and neural correlates.

If the proposers were driven primarily by strategic motives in our modified UG, as suggested in the literature (e.g. refs 8, 17, 18), their proposed offer would be altered by the paired selfish offer given the corresponding possibility of rejection and the potential monetary reward. A very selfish offer had a higher possibility of rejection than a fair offer; therefore, proposers might anticipate rejection when choosing between a fair and a very selfish offer and be inclined to choose the fair offer without much hesitation. In contrast, a slightly selfish offer is more amicable and acceptable to recipients than a very selfish offer, even though it also favours the proposers. Therefore, proposers might consider choosing the slightly selfish offer because it is more lucrative than a fair offer but somehow acceptable. Specifically, we expected that proposers would hesitate more and, thus, take longer making a decision when they had to choose between a fair offer and a slightly selfish offer than if they had to choose between a fair offer and a very selfish offer. In the neural level, we expected that proposers would show greater activation in the medial prefrontal cortex (mPFC), which has been reported to be involved in social decision making^25, 26^. Specifically, we expected that the dorsal anterior cingulate cortices (dACC; Brodmann areas [BA] 24 and 32) would be more activated as it has been found to play a central role in decisions about the allocation of cognitive control based on a cost/benefit analysis (review see ref. 27).

We expected to observe greater activation additionally in the mesolimbic region (i.e. ventral tegmental area [VTA] in the midbrain, ventral striatum [vST] and their reciprocally connected frontal cortex) signalling the greater monetary reward when proposers chose the slightly selfish offer over the fair offer^28–30^. Such risk-taking behaviour might be reflected from greater activation in the nucleus accumbens (NAcc) of the vST^31^, the insular cortex^32, 33^ and the orbitofrontal cortex^34^ as reported in economic decision-making studies.

It has been suggested that proposers are more risk averse and more sensitive to the risk of rejection when stakes are higher due to the greater potential monetary gains and losses^3, 35^. Thus, we expected that proposers would take longer to consider the recipient’s answer even though they eventually chose the fair offer in a higher stakes situation. Particularly, we expected to observe greater activation in mesolimbic region signalling with greater potential monetary gains and losses and in regions that previously have been implicated in risk-averse attitudes, such as the right dorsal lateral prefrontal cortex^36, 37^. We expected that the anterior cingulate gyrus (ACCg) would also activate more as it has been found to associate with the value of rewards for others^25, 38, 39^. Moreover, it would be of great interest to examine whether proposers are more likely to spend a shorter amount of time choosing the fair offer over a more selfish offer, particularly when the stakes are higher.

In fact, the UG proposing behaviour has been rarely studied compared to the responding behaviour. It has been commonly assumed that rejection of selfish offers by recipients reflects negative emotional arousal mediated by the anterior insular cortex (AIC) and ACC^40–43^. Thus, the findings of the current study will be a great contribution to the existing literature and could help elucidate the neural correlates of the proposing behaviour as revealed by previous behavioural research (e.g. refs 3–6).

## Results

See Methods for details of participants’ inclusion for the behavioural and imaging data analyses.

### Overall choices with different share and stake sizes: estimated proportion of fair offers

**Figure 1:**
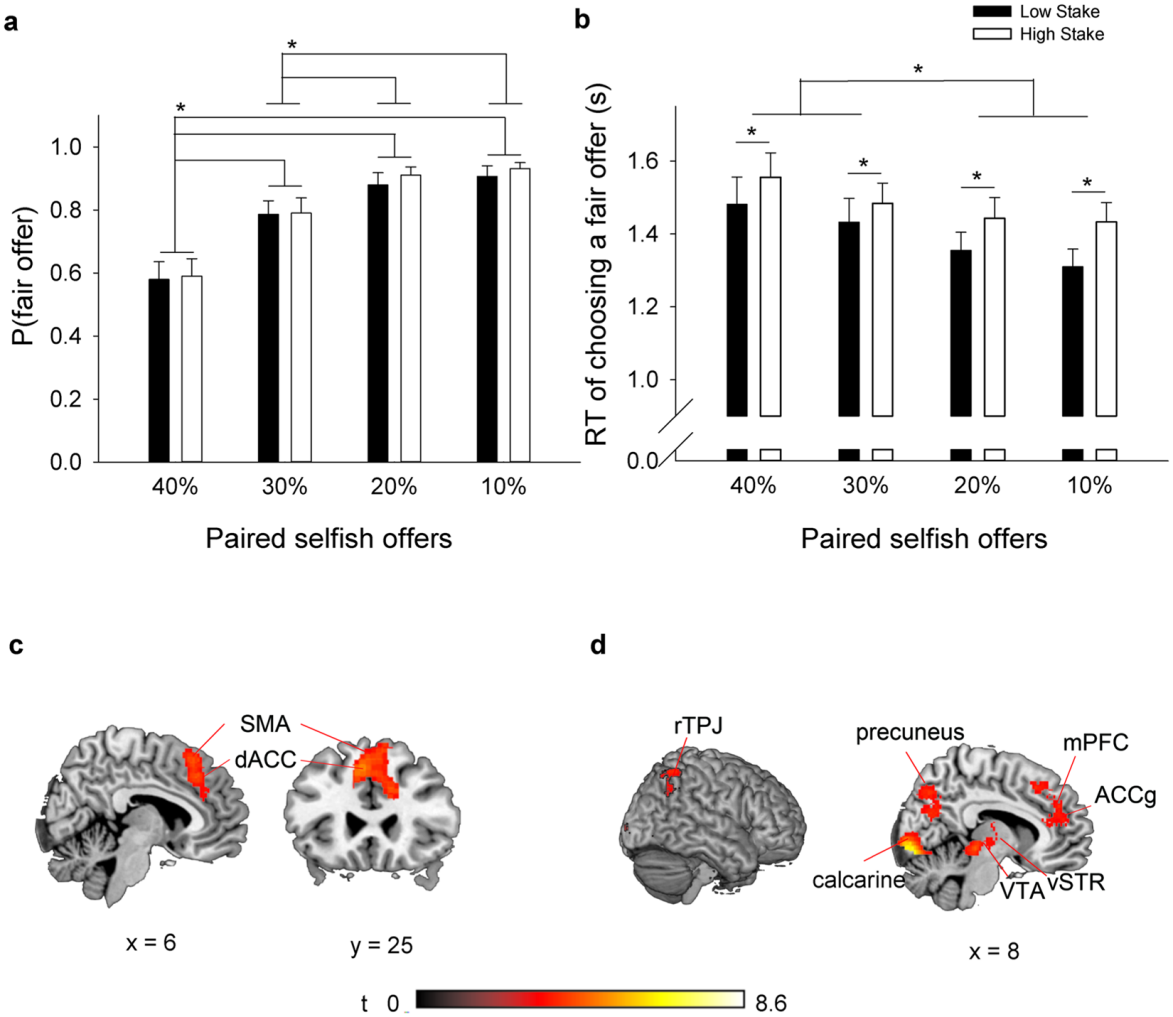
Effect of share size and stake size when choosing a fair offer. (**a**) Estimated proportion of fair offers; (**b**) average reaction times when proposers chose a fair offer over a selfish offer with different share sizes (i.e., offering 40%, 30%, 20%, and 10% of the stake) in different stakes. Error bars indicate standard errors. *p < 0.05; (**c**) significantly greater activation of a fair offer paired with a slightly selfish offer (i.e., offering 40% or 30%) than a very selfish offer (i.e., offering 20% or 10%), and (**d**) in high stakes than in low stakes.

The estimated logistic model showed a significant main effect of share size, *χ* ^2^ (3, 42) = 53.522, *p* < 0.001. Participants were significantly more likely to choose fair offers when the other choice was more selfish (estimated proportion of 0.59 ± 0.049, 0.79 ± 0.037, 0.90 ± 0.024 and 0.92 ± 0.019 when the other choice offered 40%, 30%, 20% and 10% of the stakes, respectively). Post-hoc analyses indicated that all pairs of comparisons were significant (odds ratios were 0.106 and 0.280 for the comparisons between 40% and 10% and between 30% and 10%, respectively), except when the other choice offered 20% vs. 10% of the stakes (odds ratio = 0.753). The main effect of stake size, *χ* ^2^ (1, 42) = 0.302, *p* = 0.583 (odds ratio = 0.718), and the interaction, *χ* ^2^ (3, 42) = 2.430, *p* = 0.488, were not significant (see Fig. 1a).

### Choice of a fair offer over a selfish offer with different share and stake sizes

#### Behavioural results: response time (RT) of choices for a fair offer over a selfish offer with different share and stake sizes

**Figure 2:**
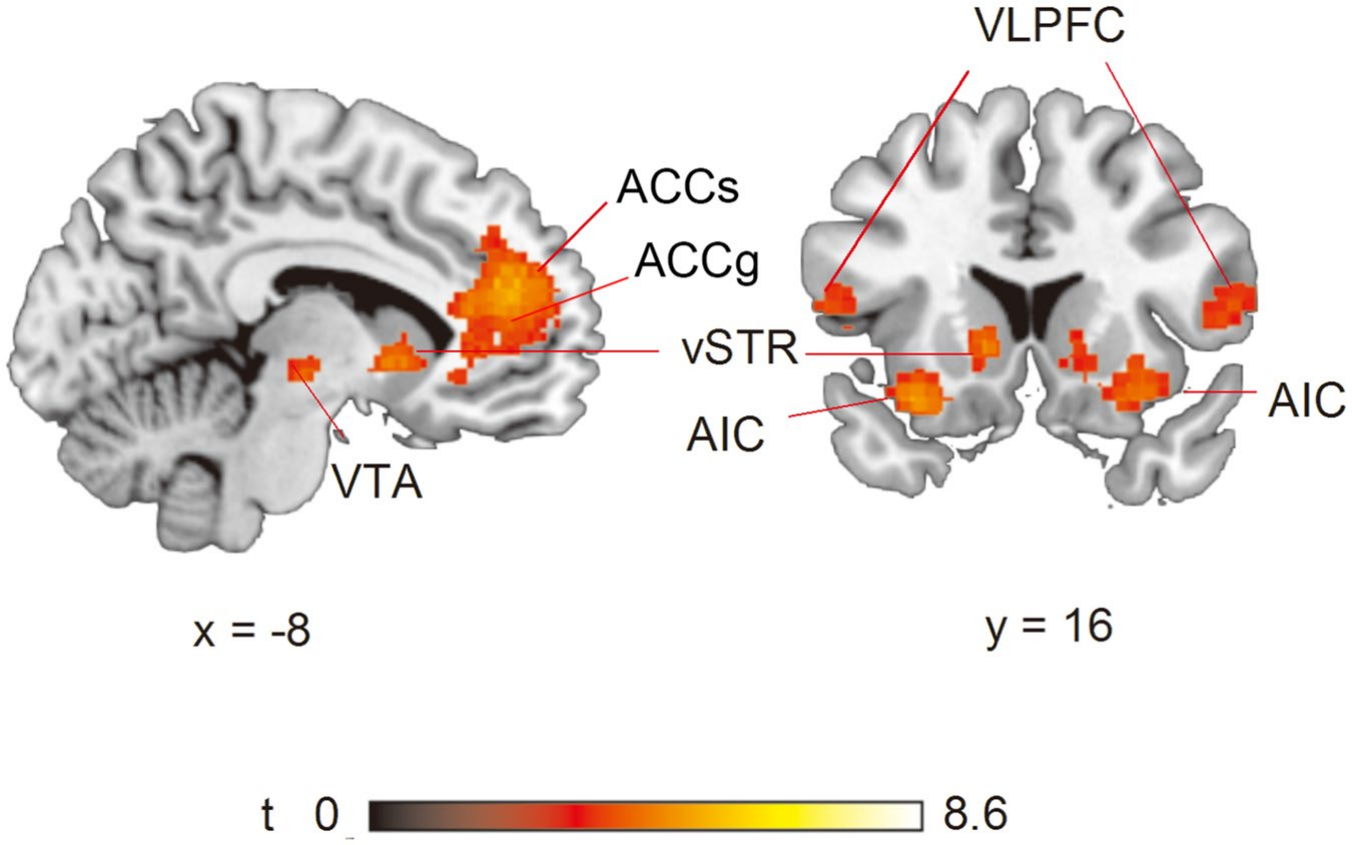
Effect of choice. Significantly greater activation induced when proposers chose a slightly selfish offer over a fair offer.

Similarly, the analysis of variance (ANOVA) detected a significant main effect of share size, *F* (1.742, 64.458) = 11.600, *p* < 0.001, *η* ^2^ = 0.239. Proposers decreased the RT of choosing a fair offer when the other choice was more selfish (mean times, 1.591 ± 0.065, 1.458 ± 0.059, 1.399 ± 0.051 and 1.371 ± 0.047 s when the other choice offered 40%, 30%, 20% and 10% of the stakes, respectively). Post-hoc analyses indicated that all compared pairs were significant, except when the other choice offered 20% vs. 10% of the stakes and when the other choice offered 40% vs. 30% of the stakes. The main effect of stake size was also significant, *F* (1, 37) = 7.923, *p* < 0.01, *η* ^2^ = 0.176. Proposers took longer for higher stakes, with mean values of 1.395 ± 0.056 and 1.479 ± 0.054 s for low and high stake sizes, respectively. No significant interaction was found, *F* (1.893, 70.057) = 0.885, *p* = 0.412, *η* ^2^ = 0.023 (see Fig. 2b). A trend analysis showed that the relationship between RT and share size of the selfish offer could be modelled by a linear trend, *F* (1, 37) = 16.378, *p* < 0.001, *η* ^2^ = 0.307.

#### Imaging results

First, choice of a fair offer over a slightly selfish offer induced greater activation in the medial frontal gyrus, including the bilateral supplementary motor areas (SMA) and the dACC than choices of a fair offer over a very selfish offer (Table 1 and Fig. 1c). The reverse contrast did not reveal any significant activation clusters. Second, choosing a fair offer with higher stakes induced greater activation in distributed regions that were more associated with two circuits, such as the mesolimbic system, including the VTA (or the substantia nigra [SN]) in the midbrain and the left pallidum in the vST and the theory of mind (ToM)^34, 44–47^, including the mPFC, bilateral precuneus and right temporoparietal junction (TPJ). In particular, the activated cluster in the mPFC was mostly in the ACCg. Additional clusters were also observed in bilateral occipital lobes and the left middle frontal gyrus (MFG)/precentral gyrus (Table 1 and Fig. 1d). The reverse contrast and the interaction effects did not reveal any significant activation clusters.

**Table 1:** Brain regions showing greater activation in choices of a fair offer over a slightly selfish offer (i.e., offering 40% or 30%) than the same choices over a very selfish offer (i.e., offering 20% or 10%); and in choices of a fair offer in higher stakes thank low stakes (*p* < 0.001 uncorrected with FDR correction at the cluster level; BA, Brodmann’s area).

Brain regions	Peak MNI x y z	t-Value	Cluster size		
**Choices of a fair offer over a slightly selfish offer > choices of a fair offer over a very selfish offer**					
Left Supplementary motor area (BA 8)	−4	24	46	5.03	1185
Left Medial frontal gyrus (BA 8)	−4	26	52	4.82	
Right Supplementary motor area (BA 8)	6	22	56	4.26	
Left Anterior cingulate gyrus (BA 32)	−10	34	26	3.88	
Right Anterior cingulate gyrus (BA 32)	6	34	28	3.44	
**Choices of a fair offer in high stakes > Choices of a fair offer in low stakes**					
Left Calcarine sulcus (BA 17)	−14	−94	−2	8.67	1832
Right Lingual gyrus (BA 17)	12	−86	−6	8.18	
Midbrain	4	−36	−8	4.88	1009
Right Midcingulate area (BA 23)	2	−12	30	4.27	
Left Pallidum	−12	4	−2	3.50	
Left Precentral gyrus (BA 6)	−34	0	52	4.67	527
Left Middle frontal gyrus (BA 4)	−28	0	52	4.33	
Right Precuneus (BA 31)	16	−62	32	4.57	1238
Right Cuneus (BA7)	16	−68	38	4.30	
Right Temporoparietal junction (BA 7)	38	−58	52	3.88	469
Right Temporoparietal junction (BA 40)	48	−54	54	3.75	
Left Anterior cingulate gyrus (BA 32)	−2	42	12	4.12	714
Right Supplementary motor area (BA 8)	4	24	48	3.88	
Right Anterior cingulate gyrus (BA 9)	10	40	26	3.83	
Right Medial frontal gyrus (BA 8)	4	28	50	3.80	

### Choosing between a fair and a slightly selfish offer with different stake sizes

#### Behavioural results

Proportion of fair offers with different stake sizes: Proposers showed similar rates when choosing a fair offer with low and high stake sizes, *t*_(27)_ = −0.174, *p* = 0.863, *r* = 0.03, with mean rates of 60% and 62% for low and high stake sizes, respectively.

RT of choices for a fair and a slightly selfish offer with different stake sizes: ANOVA detected no significant main effect of choice, *F* (1, 27) = 1.749, *p* = 0.197, *η* ^2^ = 0.061, or stake size, *F* (1, 27) = 0.328, *p* = 0.572, *η* ^2^ = 0.012, and no significant interaction, *F* (1, 27) = 1.530, *p* = 0.227, *η* ^2^ = 0.054, with mean values of 1.592 ± 0.079, 1.654 ± 0.072, 1.704 ± 0.077 and 1.678 ± 0.094 s for choosing a fair offer with low stakes, a fair offer with high stakes, a slightly selfish offer with low stakes and a slightly selfish offer with high stakes, respectively.

#### Imaging results

As shown in Table 2 and Fig. 2, choosing a slightly selfish offer induced greater activation in the mPFC than choosing a fair offer (mainly in the ACCg and partially in the anterior cingulate sulcus [ACCs]), and regions associated within the mesolimbic system, including the VTA (or the SN) in the midbrain and caudate and pallidum and nucleus accumbens (NAcc) in the vST. The bilateral AIC and ventrolateral prefrontal cortex (VLPFC), including the inferior frontal gyrus and precentral gyrus also displayed greater activation. The other contrasts and the interaction effects did not reveal significant activation clusters.

**Table 2:** Brain regions showing greater activation in choices of a slightly selfish offer (i.e., offering 40% or 30%) than a fair offer (*p* < 0.001 uncorrected with FDR correction at the cluster level; BA, Brodmann’s area).

Brain regions	Peak MNI			t-Value	Cluster size
	x	y	z		
Right Midbrain	6	−22	−8	5.60	404
Left Medial frontal gyrus (BA 9)	−8	44	18	5.25	2794
Left Anterior cingulate gyrus (BA 9)	−8	40	22	5.23	
Right Anterior cingulate gyrus (BA 32)	14	40	16	5.09	
Left Insula	−32	16	−16	5.43	380
Right Caudate (body)	12	10	−6	4.85	213
Right Insula(BA 47)	26	16	−16	4.33	421
Left Inferior frontal gyrus (opercular part; BA 44)	−48	8	14	4.77	330
Left Precentral gyrus (BA 6)	−54	2	18	4.11	
Left Caudate (head)	−12	16	0	4.76	186
Left Pallidum	−14	4	−6	3.45	
Right Inferior Frontal Gyrus (triangular part; BA 45)	56	24	10	4.43	505
Right Precentral gyrus (BA 9)	36	4	30	3.87	

## Discussion

Proposers typically make a fair offer in the original UG^3, 4, 6^, and our proposers also showed extremely high rates of choosing the fair offer when the other choice was very selfish in our modified UG. In contrast, when the choice was only slightly selfish, suggesting comparatively lower rejection risk than a very selfish offer, and the higher expected monetary reward than a fair offer, but somehow acceptable, the proposers showed much lower rates of choosing the fair offer. This result was consistent with previous findings^14^. Moreover, our systematic manipulation of the share size of the paired selfish offer allowed us to investigate the rate of choosing a fair offer as share size of the paired selfish offer changes. We found that proposers increased their rates of choosing a fair offer as share size of the paired selfish offer decreased and, more importantly, they showed similar rates when the share sizes were 20% and 10%. Thus, a selfish offer, such as 20%, could be a threshold at which proposers no longer consider the offer, which corresponded perfectly with the threshold that recipients typically reject in the classical UG (e.g. ref. 4). This result suggests that proposers in our modified UG were putting themselves in the shoes of the recipient, and the format of our modified UG retained the essence of the original UG.

Furthermore, the finding that proposers increased RT when choosing a fair offer as a function of share size of the paired selfish offer was new. Moreover, they spent similar time choosing a fair offer when the selfish offer was 40% vs. 30% and 20% vs. 10%. This result further provides justification for the way that we merged the imaging data of 40% and 30% as ‘slightly selfish offers’ and 20% and 10% as ‘very selfish offers’. When choosing between the fair and slightly selfish offers than a very selfish offer, proposers’ dACC was more activated. The dACC has been shown to relate to the allocation of cognitive control in making decisions considering the potential gains and losses^27^. Moreover, it has been found to frequently engage in pre-response conflict and decision uncertainty that signals a reduced possibility of obtaining an anticipated reward^48–51^. Furthermore, it has also been shown to signal a foregone reward for self^25, 38^. We also found greater activation in the rostral cingulate zone (BA 32), which has been shown to have direct and indirect projections and functional connectivity to the SMA and activate more when choosing between competing options in consideration of an anticipated reward associated with each of the options^51–53^. Similarly, we found greater activation in the SMA, which has been reported to engage in motor control during goal-based action selection processes (i.e. choosing between a fair offer and a selfish offer)^51–53^.

An important contribution of this study was that we examined the behavioural and neural responses when proposers made a slightly selfish offer over a fair offer (as in the second subset of data analyses), which has never been reported in the literature. Proposers did not have different decision times when choosing between a fair and a slightly selfish offer or between splitting high and low stakes. The latter result was particularly interesting because proposers took longer for higher stakes when they chose a fair offer (as shown in our first subset of data analyses). The stake effect diminished when proposers chose the slightly selfish offer, suggesting that proposers may have used different considerations when making fair and selfish offers. However, the different sample sizes and data inclusion between the two subsets of data analyses could also make this difference.

Choosing a slightly selfish offer induced greater activation in the mPFC than choosing a fair offer (mostly in the ACCg and partly in the ACCs), implying a greater consideration of the rewards for one self and the other (recipient) (e.g. refs 25, 38, 39, 54, 55). Regions within the mesolimbic system (including the VTA or the SN in the midbrain and the left pallidum in the vST) were also more activated, possibly signalling the potential higher value when choosing the selfish offer (e.g. ref. 28).

Moreover, greater activation was also found in the bilateral AIC. It has been suggested that AIC functions should be inferred with co-activated regions because the AIC is associated with a broad range of functions linked to emotions and homeostatic regulation^33, 56–59^. The co-activated regions involved in the reward circuit imply representation of reward anticipation by the AIC^60, 61^. Moreover, co-activated regions, such as NAcc in the vST, imply that the activation in the AIC might be related to the representation of risk level for a slightly selfish offer because it was more likely to be rejected than a fair offer^32, 33, 62, 63^. Alternatively, greater activation in the AIC might also associate with negative emotion expression or regulation due to the consideration of choosing a slightly selfish offer over a fair offer. Such an interpretation addresses processing of negative emotion as reported by Sanfey *et al*.^42^ that UG recipients display greater activation in their bilateral AIC for an unfair offer than a fair offer, as a symbol of negative emotion, such as disgust at a disrespectfully selfish offer. Last, the higher activation in the VLPFC might be related to cognitive control and response inhibition because choosing a slightly selfish offer was more rewarding but also riskier than choosing a fair offer^64, 65^. It would be interesting for future studies to use the AIC as a region of interest to investigate the psychological processing of proposers and recipients to better understand their different concerns, such as self-interest and fairness.

In this study we also examined the effect of stake size, and not surprisingly, we replicated previous findings that stake size did not significantly alter the proposed offer (e.g. ref. 3). Moreover, we provided new evidence that proposers took longer to choose the fair offer when the stakes were higher. It is also possible that proposers took longer when the stakes were higher because higher stakes involve more math skills (e.g. to calculate the percentage of the selfish offer to total stakes). However, after cancelling out the effect of RT in our imaging data with the parametric modulation analysis, proposers still showed greater activation in the brain regions that have been previously shown to associate with reward and ToM^44–47^. Notably, higher stakes also elicited greater activation in the ACCg, which has been reported to signal rewards delivering to others^25, 38, 39, 54, 55^. Taken together, these imaging results might imply that proposers deliberated over the recipients’ answers to a greater extent when stakes were higher due to the higher potential gains and losses. One limitation of the current study was that stake size may not have been high enough to make a difference on choices. Future studies should test proposing behaviour changes when stake size is extremely high.

It is worthwhile to compare our results with previous meta-analyses findings of responding behaviour during the UG^66^. Previous studies reported that recipients showed greater activation in the bilateral mid-anterior insula, bilateral anterior midcingulate cortex (aMCC), left anterior SMA and right cerebellum when facing an unfair compared to a fair offer. They also indicated that recipients had greater activities in the SMA, aMCC, right MFG and bilateral lentiform nucleus when rejecting an unfair offer rather than when they accepted an offer. Activation in the mPFC/ACC while proposing a fair offer and rejecting an unfair offer is in line with the view that this region reflects rewards of decisions during social interactions^25, 54, 55^.

The present study complemented the current literature as there are only two fMRI studies that investigated UG proposing behaviours to any extent^13, 14^. Weiland *et al*.^13^ attempted to investigate UG proposing behaviour but were constrained by the very unbalanced number of choices between fair and selfish offers. The division of fair and selfish offers could also be debated because the offer of 7:5 was considered the fair offer when contrasting fair offers (i.e. offers of 6:6 and 7:5) vs. selfish offers (i.e. offers of 8:4, 9:3, 10:2 and 11:1). Moreover, their UG data were obtained from only 14 participants. Therefore, their results should be interpreted with caution. Furthermore, Zheng and Zhu^14^ did not compare fair and selfish offers.

In conclusion, in our modified UG, proposers were less likely and spent more time choosing the fair offer over a slightly-selfish offer than a very selfish offer independent of stakes, possibly because a slightly selfish offer was more lucrative than a fair offer but somehow acceptable to the recipient. Such choices induced greater activation in the dACC that typically engage in allocation of cognitive control for cost/benefit decision making. Furthermore, choosing a fair offer in higher stakes evoked greater activation in the ACCg that signal the rewards for the other person and the areas that previously have been implicated in reward and ToM. These results might imply that in higher stakes participants deliberated on recipients’ answers due to higher potential gains and losses. Last, choosing a slightly selfish offer over a fair offer evoked greater activation in the ACCs, ACCg, VTA (or SN), and AIC, tracking rewards for oneself and the other person as well as signalling a higher value and a greater rejection risk In sum, we have provided a comprehensive picture of UG proposing behaviour from various aspects. Our behavioural and imaging findings favoured the hypothesis of strategic motives for fair UG offers, which assumes that a fair offer is made to avoid rejection and receive reward.

## Methods

### Participants

Forty-five participants (27 females and 18 males; mean age = 24.49 ± 2.70 years) were recruited. All participants were healthy, right-handed (assessed by the Edinburgh Handedness Inventory; Oldfield 1971, see ref. 67) and without any neurological or psychiatric disorders or contraindications to MRI. They were matched with another 45 participants who acted as recipients to accept or reject their proposals. Prior to the experiment, all participants gave written informed consent to the study. All methods were performed in accordance with the ethical principles of the Declaration of Helsinki and were approved by the institutional review board on Humanities & Social Science Research/IRB-HS at Academia Sinica.

### Task, Stimuli and Procedure

We explained our modified UG task to the participants before testing, and the participants completed eight practice trials. We informed the participants that another participant would be selected randomly to play the role of recipient and respond to their offers (160 trials in total) in the UG in the other study. Participants did not meet the recipients and did not know who they would be matched with.

We told participants that two trials would be selected randomly out of all testing trials and that they would receive money based on the matched recipients’ answers to their proposed offer. The average amount of money that they received from the two selected trials was NT$901 ± NT$452 (range, NT$0 – NT$1740; exchange rate between NT$ and US$ was approximately 33:1). This amount of money was approximately eight times the minimum wage per hour in Taiwan. Because most of the participants were college students, we assumed that such an amount could be an incentive for them to get as much money as possible. Moreover, they received NT$500 as a participation fee.

**Figure 3:**
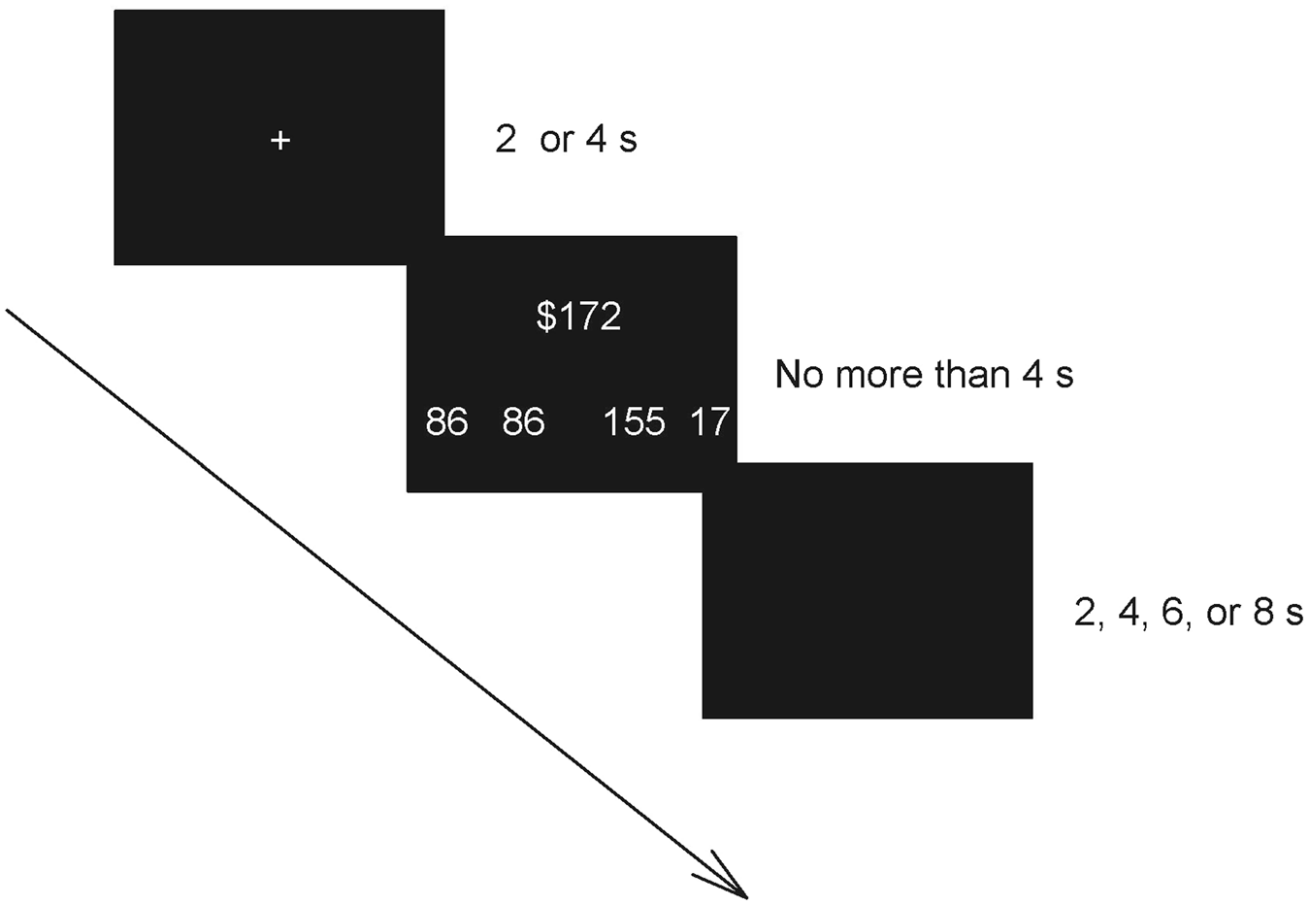
Timeline of an exemplar trial. In each trial, participants had to choose between a fair offer and a very selfish offer (i.e., offering 10% of the stake to the recipient) in a low stake.

Participants were presented with a fixation cross for 2 or 4 s during each trial, and then a stake with two pairs of choices of a fair offer and a selfish offer. Participants had to choose one pair of choices by pressing the button with their left or right thumb in 4 s; otherwise the trial would be skipped. No feedback was given, so we could observe participants’ original proposing behaviour without being influenced by the immediate answer of the recipient. The jittered inter-trial interval (ITI) was 2, 4, 6, or 8 s (Fig. 3). The high stakes ranged from NT$1640 to NT$2360 with an average of NT$2000 and a coefficient of variation (CV) of 0.098. The low stake ranged from NT$164 to NT$236 with an average of NT$200 and the same CV value. The fixed CV of the two stake sizes ensured that the differences among the four levels of the share size were consistent for the two stake sizes. The fair offer was offering 50% of the stake to the recipient, and the selfish offer was offering 40%, 30%, 20% or 10% of the stake. The position (left vs. right) of the fair vs. selfish offer choices was randomised, and the position (left vs. right) of the money assigned to the two players in the offer choice was counterbalanced among participants. Each experimental condition (2 stake sizes × 4 share sizes) was tested in 20 repetitions, yielding a total of 160 trials divided into four runs. Participants also received a 6 min anatomical scan. The entire experiment took approximately 1 hr. The experimental program was written using MATLAB2008 (MathWorks Inc., Natick, MA, USA) with Psychotoolbox 2.5.4. The choices, RTs, and brain images were recorded.

### Data Acquisition

MRI images were collected using a 32-channel head coil in a 3T scanner (Skyra, Siemens Medical Solutions, Erlangen, Germany). A T2*-weighted gradient-echo echo planar imaging sequence was used for fMRI scanning, with 3 mm slice thickness, 256 × 256 mm^2^ field of view, 90° flip angle, 34 slices, 2000 ms repetition time (TR), and 30 ms echo time (TE). The anatomical, T1-weighted high-resolution image (1 × 1 × 1 mm^3^) was acquired using a standard MPRAGE sequence, with a 7° flip angle, 2,530 ms TR, 3.3 ms TE and 1,100 ms inversion time (TI).

### Data Analysis

Among the 45 participants, two (participants 18 and 34) were excluded from the data analysis because they fell asleep in the MRI scanner during the experiment. For imaging analysis, only participants without excessive head movement (i.e. overall motion <3 mm across the runs and <2 mm of motion between adjacent functional volumes) were included.

### Overall choices and RTs with different share and stake sizes

Forty-three participants (25 females and 18 males; mean age = 23.67 ± 3.00 years) were included in this behavioural data analysis.

We analysed the UG binary choices using repeated-measures logistic regression implemented with the generalized estimating equations method. We modelled the within-subject effects of share size, stake size and their interaction for the fair offers. We also analysed RTs using repeated-measures ANOVA with share size and stake size as within-subject factors (see Supplementary Information for the RT results). IBM SPSS 20.0 was used for the statistical analysis (IBM Corp., Armonk, NY, USA) with the α value set at 0.05. In situations in which sphericity was violated, we employed the Greenhouse-Geisser correction. Bonferroni’s correction was used for post-hoc multiple comparisons. All behavioural data were analysed with the same criteria.

Crucially, as found robustly in the classical UG that the majority of proposers offered fairly (e.g., ref. 4), we examined the choices of a fair offer over a selfish offer with different share sizes and different stake sizes to understand the proposing behaviour in our modified UG in the first subset of data analyses. In addition to the behaviour of offering fairly, we were also interested in the behaviour of offering selfishly. Consistent with the literature^3–5^, the possibility of making a very selfish offer was very low and that made for very few corresponding images and unreliable imaging results (see Supplementary Information Table S1 for details). Therefore, we selected only the cases in which proposers had to choose between a fair offer and a slightly selfish offer (i.e. offering 40% or 30% of the stake) in different stake sizes for a second subset of data analyses.

### Choices of a fair offer over a selfish offer with different share and stake sizes

Of the 43 participants, five (participants 24, 43, 45, 49 and 59) were not included because they did not have data for certain conditions, resulting in 38 participants included in the RT analysis of fair offers.

We compared the RTs of choosing a fair offer over a selfish offer with four different share sizes and two stakes in a two-way (4 share sizes × 2 stake sizes) repeated-measures ANOVA with share size and stake size as within-subject factors. Furthermore, we tested the trend model that best explained the RTs for choosing a fair offer contingent on share size of the paired selfish offer using polynomial contrasts.

Five participants (participants 38, 39, 55, 57 and 60) were excluded from the imaging analyses, given extensive head motion, and another six participants (participants 24, 43, 45, 46, 49 and 59) were excluded because they did not have sufficient trials per condition (n < 5) to gain adequate statistical power (see Supplementary Information Table S1 for details). Consequently, 32 participants (19 females and 13 males; mean age = 23.97 ± 3.12 years) were included. The 32 participants showed the same tendency in their behavioural data (both the proportion and RT of fair offers) as the 43 participants.

Imaging analysis was performed using the Statistical Parametric Mapping 8 (Wellcome Trust Centre for Neuroimaging, London, UK) software package. The functional images of each participant were corrected for slice timing and head motion and then co-registered to the participant’s segmented grey matter image. Next, the images were normalized to the Montreal Neurological Institute (MNI) standard space and spatially smoothed by convolution using an 8 mm full width at half maximum Gaussian kernel. For simplicity and to ensure sufficient statistical power for the imaging analysis, we merged the options that offered 40% and 30% of the stake as ‘slightly selfish offers’, and options that offered 20% and 10% of the stake as ‘very selfish offers’. Consequently, we obtained up to nine different response conditions from the participant’s choice (a fair or a selfish offer) in the four experimental conditions (2 merged share sizes × 2 stake sizes) plus one error response condition in which participants did not make a choice within 4 s. We modelled the data on each participant with up to nine regressors using the general linear model as the first-level analysis. Next, we specified the onset and duration (0 s) of each response trial and entered the corresponding RT as the parametric modulator with first-order modulation to avoid RT variability correlated with the blood-oxygenation-level-dependent signal^68^. The six parameters of the realignment were also included in the model as regressors of no interest. The parameter estimates for choosing a fair offer by each participant were fed into a two-way (2 merged share sizes × 2 stake sizes) flexible factorial design with merged share size and stake size as within-subject factors using a random-effects analysis for the second level. The t-contrasts of interest were the differences in merged share sizes and stake sizes and the interaction effects. The threshold of the statistical maps was at a voxel-wise intensity of *p* < 0.001 (uncorrected) with a false discovery rate correction at the cluster level using the whole brain as the volume of interest. The resulting regions of activation were characterized in terms of their peak voxels in the MNI coordinate space.

### Choosing between a fair and a slightly selfish offer with different stake sizes

Of the 43 participants, nine (participants 17, 23, 25, 27, 35, 38, 39, 55 and 60) always chose fair offers and six participants (participants 20, 28, 29, 45, 49 and 59) did not have data under certain conditions (see Supplementary Information Table S1 for details). Therefore, 28 participants (18 females and 10 males; mean age = 23.79 ± 3.10 years) were included in this behavioural analysis.

The rates of choosing a fair offer in the different stake sizes were compared using a paired *t*-test. The RT between choices of a fair and a slightly selfish offer in different stake sizes was compared using a two-way (2 choices × 2 stake sizes) repeated-measures ANOVA with choice and stake size as within-subject factors.

Of the 28 participants, one (participant 57) had extensive head motion and eight (participants 24, 30, 31, 33, 40, 43, 46 and 56) did not have sufficient images per condition (n < 5) (see Supplementary Information Table S1 for details). In the end, 19 participants (12 females and 7 males; mean age = 24.84 ± 3.22 years) were included in the imaging analysis. The 19 participants showed a similar pattern in their behaviours as the 28 participants included in the behavioural analysis.

The parametric estimates for each participant (i.e. choices of a fair offer and a slightly selfish offer in low and high stakes) obtained in the first-level analysis were entered into a two-way (2 choices × 2 stake sizes) flexible factorial design with choice and stake size as within-subject factors in a random-effects group-level analysis for the imaging analysis. The t-contrasts of interest were the differences in choices and stake sizes and the interaction effects. The significance levels for behavioural and imaging analyses were identical to those in the first subset of data analyses.

## Electronic supplementary material

Supplementary information
