# Supplementary material for: Strategic Motives Drive Proposers to Offer Fairly in Ultimatum Games: An fMRI Study
Source: Sci Rep. 2017 Apr 3;7:527. doi: 10.1038/s41598-017-00608-8 (PMC5428836; doi:10.1038/s41598-017-00608-8)
Supplement: Supplementary file 1 — Supplementary information [file 41598_2017_608_MOESM1_ESM.pdf]

Strategic Motives Drive Proposers to Offer Fairly in Ultimatum Games: An fMRI Study

Yin-Hua Chen, Ying-Chun Chen, Wen-Jui Kuo, Kamhon Kan, C. C. Yang, & Nai-Shing Yen

Supplementary Information

Results

Overall RTs with different share and stake sizes

The repeated-measures analysis of variance (ANOVA) detected a significant main effect of share size, F (1.994, 83.741) = 13.726, $p < .001$, $\eta^2 = .246$. Proposers decreased the RT of choosing a fair offer when the other choice was more selfish (mean times, 1.539 ± .058, 1.523 ± .061, 1.453 ± .056 and 1.416 ± .050 s when the other choice offered 40%, 30%, 20%, and 10% of the stakes, respectively). Post-hoc analyses indicated that all pairs of comparisons were significant, except when the other choice offered 40% vs. 30% of the stakes and when the other choice offered 20% vs. 10% of the stakes. The main effect of stake size was also significant, F (1, 42) = 12.596, $p = .001$, $\eta^2 = .231$. Proposers took longer for higher stakes, with mean values of 1.443 ± .055 and 1.523 ± .057 s for low and high stake sizes, respectively. No significant interaction was found, F (2.393, 100.519) = 2.738, $p = .060$, $\eta^2 = .061$.

Table S1. The number of choices of a fair and a selfish offer (choices taken were highlighted in bold) and error trials from all participants.

participant # / conditions	P16	P17	P19	P20	P21	P22	P23	P24	P25	P26	P27	P28	P29	P30	P31	P32	P33	P35	P36	P37	P38	P39	P40	P41	P42	P43	P44	P45	P46	P47	P48	P49	P50	P51	P52	P53	P54	P55	P56	P57	P58	P59	P60
200<br>**50%** vs. 40% or 30%	19	40	22	40	11	22	40	23	40	16	40	33	40	22	38	30	38	40	31	24	40	40	30	29	24	3	12	2	3	21	34	0	27	20	33	30	26	40	37	12	33	19	40
200<br>**50%** vs. 20% or 10%	40	40	40	40	34	38	40	40	40	38	40	39	40	40	39	39	38	40	36	38	40	40	31	37	40	2	37	37	6	28	40	3	40	40	34	39	40	39	39	23	37	39	40
2000<br>**50%** vs. 40% or 30%	13	40	35	39	18	18	40	1	40	28	40	40	23	37	39	22	18	40	10	27	40	40	37	34	26	39	8	0	35	22	16	35	29	21	25	27	25	40	39	12	31	0	40
2000<br>**50%** vs. 20% or 10%	36	40	39	40	38	39	40	33	39	40	40	40	40	40	40	38	26	40	36	39	40	40	37	38	40	38	25	36	36	25	24	40	40	40	31	37	40	39	40	23	40	24	40
200<br>50% vs. **40% or 30%**	21	0	18	0	29	18	0	17	0	24	0	2	0	18	1	10	2	0	9	16	0	0	10	11	15	37	27	38	37	21	6	40	13	20	6	10	14	0	3	27	7	21	0
200<br>50% vs. **20% or 10%**	0	0	0	0	5	2	0	0	0	2	0	0	0	0	1	1	2	0	4	2	0	0	9	3	0	38	3	3	34	13	0	37	0	0	4	1	0	0	1	16	3	1	0
2000<br>50% vs. **40% or 30%**	27	0	5	1	21	22	0	39	0	12	0	0	17	3	1	18	22	0	30	13	0	0	3	6	14	1	32	40	5	16	24	5	10	19	14	13	14	0	1	28	9	40	0
2000<br>50% vs. **20% or 10%**	3	0	1	0	2	1	0	6	0	0	0	0	0	0	0	0	14	0	4	1	0	0	3	2	0	2	15	3	4	14	15	0	0	0	8	3	0	0	0	16	0	16	0
Error	1	0	0	0	2	0	0	1	1	0	0	6	0	0	1	2	0	0	0	0	0	0	0	0	1	0	1	1	0	0	1	0	1	0	5	0	1	2	0	3	0	0	0
